# Introduction of a novel frequency converter using piezoelectric vibrations modes

**DOI:** 10.1038/s41598-023-38139-0

**Published:** 2023-07-07

**Authors:** Ava Pirayande, Yousef Hojjat

**Affiliations:** Department of Mechanical Engineering, Tarbiat Modares University, Tehran, Iran

**Keywords:** Mechanical engineering, Electrical and electronic engineering

## Abstract

Converting the frequency is needed in many fields of advanced technology. “Electric circuits” or “coupled motors and generators” are usually used for frequency conversion. This article introduces a new piezoelectric frequency converter (PFC), using an idea similar to piezoelectric transformers (PT). PFC uses two piezoelectric discs as input and output elements which are pushed together. There is a common electrode between these two elements and two electrodes input and output on the other sides. When the input disc is forced to vibrate in the out-of-plane mode, the output disc vibrates in its radial mode. By applying different input frequencies, different output frequencies can be obtained. However, the input and output frequencies are limited to the piezoelectric element’s out-of-plane and radial modes. Therefore, the proper size of piezoelectric discs should be used to get the necessary gain. Simulation and experiments show that the mechanism works as predicted, and their results are in good agreement. For the chosen piezoelectric disc, the lowest gain increases the frequency from 61.9 to 118 kHz, and the highest gain increases the frequency from 3.7 to 51 kHz.

## Introduction

Piezoelectric elements have been used to make voltage transformers (PT) since a few decades ago. PTs transmit the voltage via mechanical vibration and increase or decrease it. These transformers usually consist of two piezoelectric elements as input and output ports. The input piezoelectric disc is excited at the resonance frequency, and the output voltage is obtained from the output element. The desirable ratio of input and output voltages can be obtained with proper design. Rosen first introduced PT in 1954^1^. After that, a lot of research was done on different PTs, including geometry, modeling, and efficiency improvement.

PTs are classified into three main types: Rosen, thickness vibration mode, and radial vibration mode. In the Rosen-type transformer, the input part is in the longitudinal direction and the output is in the thickness direction, and this type of PT is usually used as a voltage step-up transformer^2^. Thickness vibration mode PTs were developed by NEC of Japan in the 1990s. Radial mode devices are disc or ring-shaped and operate at a frequency close to the radial resonance. Various arrangements for the radial mode device have been proposed, of which the radial mode transformer is probably the best-known^3^. Radial mode transformers are suitable for use in up and down converters and have been used in a wide range of applications from fluorescent lamp ballasts^4^ to laptop power supplies^5^.

The coupling factor that most closely corresponds to the radial mode vibration is kp. This helps radial mode PTs to achieve high power density. Radial mode transformers have been offered at power levels of more than 100 W and are expected to exceed 200 W through further development^6^.

PTs can operate in many possible vibrational modes, each with a different frequency. However, each PT topology has an optimal vibration mode that allows optimal energy transfer. The optimal vibration mode for a piezoelectric transformer is usually the mode that has the highest electromechanical coupling and the lowest loss^7^.

Among the advantages of PTs compared to electromagnetic transformers are higher power density, absence of electromagnetic noise, higher efficiency in resonant mode, simplicity, small size, non-flammability, and simpler manufacturing process. Since PTs operate at a high frequency in their resonant frequency, piezoelectric materials should have a high mechanical quality factor and low dielectric loss at the same time^8^.

In 1992, Osamu et al. introduced a new type of multilayer PT made up of PbTiO_3_. Their goal was to use it for switching power supplies. The vibration mode was along the thickness. The electromechanical equivalent circuit showed an efficiency of more than 90%. They made the device and evaluated it experimentally. The results showed low spurious vibrations as well as good resonance characteristics at 2 MHz frequency. Also, the PT produced a power density of 16 W/cm.s without spurious vibrations. Finally, they made a high-power class E switching power supply using this PT^9^.

To solve the problem of structural damping in 2006, Guyomar et al. introduced an innovative nonlinear processing technique derived from a technique called ‘synchronized switch damping’, to avoid the strong damping of the mechanical wave of the PT and maintain the transmitted power level. Therefore, efficiency and power increase significantly^10^.

In line with this issue, Hemsel et al. introduced a new design of a single-pole transformer in 2006 that uses radial mode in input and output for 30 W power with 98% efficiency and a temperature increase of 30 °C. The characteristics described by an electromechanical equivalent circuit. Using this research, a high-power transformer suitable for various applications such as power supplies, AC/DC adapters, battery chargers, and car lighting can be obtained by connecting several multi-layer monopolar structures in parallel^11^.

To increase the efficiency and high conversion ratio, Erhart conducted experimental research in 2016 on the non-homogeneous polar disc transformer, where the input and output components were respectively in the thickness and radial directions. The optimal diameter ratio was obtained experimentally equal to 0.4. All studied disc transformers showed a very high conversion ratio (≈50) in no-load conditions and high efficiency (≈90%) in optimal load, which allows the production of plasma in the output electrode^12^.

Modit Khanna et al. in 2016 used an additional piezoelectric section (control layer) placed between the existing primary and secondary layers to have a specific voltage gain. The initial experiment device was made of discs of hard piezoelectric material and polarized in the thickness direction. They used an external variable capacitor connected to the control terminal and effectively increased the reflection capacitance and decreased the resonant frequency of the Tunable Piezoelectric Transformer (TPT). Therefore, by changing the effective value of the external capacitor in the desired range, the output voltage of TPT can be adjusted under different load conditions^13^.

In 2020, Xiao et al. designed a ring-type step-down PT based on PZT8. These transformers exhibit pure radial vibration. The minimum electrical displacement around the gap. Therefore, the PTs clearly showed optimal performance at the resonant frequency. The simulation results were in good agreement with the experimental measurements, therefore, the finite element method has a high potential in the design and analysis of PT^14^.

The review carried out by the authors shows that all the research is focused on voltage conversion (PT), and no reports on frequency converters (PFC) have been presented. The authors realized that frequency converters can also be achieved by using vibration piezoelectric discs in different modes.

For frequency conversion, it is necessary to investigate the behavior and vibration modes of piezoelectric discs. A mode is an inherent piezoelectric element vibration characteristic and each mode has its own natural frequency, damping ratio, and mode shape. Numerous experimental reports show that there are various vibration modes in piezoelectric discs. The 5 types of vibration modes identified by Ikegami et al. by finite element analysis are thickness tensile (TE), shear (T), edge (E), radial (R), and high-frequency radial (A) modes^15^. Extensive efforts have been made by Mindlin et al. since the 1950s to use classical elasticity theory to analyze the vibration behavior of discs with limited diameter-to-thickness (D/T) ratios^16,17^. In 1992, Guo et al. investigated the geometry dependence of vibration characteristics at D/T ratios of 0.1 to 20 with a finite element method and modal analysis. It was found that the vibration characteristics of a piezoelectric disc change drastically with the change of D/T. In this research, the modal constant was calculated using the modal analysis technique to obtain the strength of each resonance. The results showed that the thickness stretching mode, especially in discs with a D/T ratio greater than 5, has a much larger modal constant than other modes, and numerically and experimentally, it was shown that the modal constant approach is a suitable and reliable tool to evaluate the power of a mode stimulation^18^.

Heyliger and Ramirez in 2000 considered the free vibration of multilayer circular piezoelectric discs to calculate the natural frequencies. They introduced a numerical model that combined one-dimensional finite element approximations in the thickness direction and in-plane analytical functions^15^. Huang and Ma in 2001 investigated the out-of-plane and in-plane vibration characteristics for rectangular piezoceramic plates with completely free boundaries and showed that the out-of-plane vibration modes with low resonant frequencies cannot be measured by impedance analysis^19^.

In 2014, Huang et al. investigated the transverse and planar vibration characteristics of a two-layer piezoelectric disc for tension-free boundary conditions. In this study, they observed that the resonance frequencies and shape of the series and parallel piezoelectric disc modes show different dynamic characteristics in resonance^20^.

In this research, a converter is introduced, that multiplies the input frequencies. The converter can be a single disc or double disc. In this article, the frequency conversion phenomenon is introduced, then investigated for different piezoelectric disc sizes by the finite element method. Finally, the idea has been validated by a couple of experiments.

The introduced frequency converter can increase the frequency with a high conversion ratio, longer life, smaller size, and lower price.

## Principles and structure

**Figure 1:**
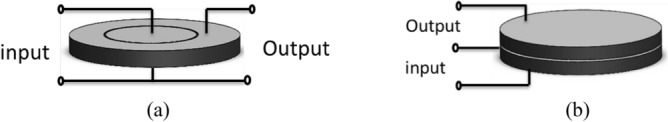
Frequency converter configurations (**a**) one disc, (**b**) two discs.

Two configurations are proposed for the piezoelectric frequency converter. One of the configurations is a piezoelectric disc with two input and output electrodes on one side (preferably concentric), and the common electrode on the other side (Fig. 1a). In the second configuration (Fig. 1b), two discs are considered, as input and output. These two discs are pressed together in a sliding state, without any intermediate material and the common electrode is between them. If these two electrodes are stuck together with glue, they will no longer be sliding and the device will not work.

In this research, both arrangements were tested. The arrangement in Fig. 1a works only at high voltages and a limited conversion ratio, which is difficult to control due to piezoelectric element brittleness feature, and lack of preload. So, the arrangement in Fig. 1b was chosen.

**Figure 2:**
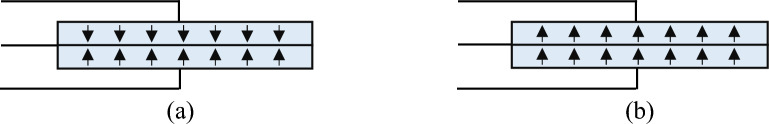
Arrangements of two piezoelectric discs on each other (**a**) series, (**b**) parallel.

In the series type, the polarizations of both piezoelectric discs are opposite (Fig. 2a), and in the parallel mode, they are identical (Fig. 2b). In this research, the series type is considered, as compared to the parallel type, its amplitude of the out-of-plane vibration is more for the same voltage. Piezoelectric disc used in this research, are two PZT4 and their properties are listed in Table 1.

**Table 1:** Properties of used piezoelectric material^21^.

Parameter	Symbol	Value
Electromechanical coupling coefficient	K_p_	0.60
	K_31_	0.36
	K_33_	0.70
	K_15_	0.70
	K_t_	0.48
Relative electric capacity rate of freedom	${\varepsilon }_{{r}^{3}}^{T}$	1350
	${\varepsilon }_{{r}^{1}}^{T}$	1900
Constant of elasticity (× 10^–12^ m^2^/N)	${s}_{11}^{E}$	13
	${s}_{33}^{D}$	8.5
	${s}_{55}^{D}$	21.5
Piezoelectricity (× 10^–12^ m/v or C/N)	d_31_	− 150
	d_33_	320
	d_15_	530
Mechanical quality factor	Q_m_	400
Frequency constant (Hz.m)	N_d_	2200
	N_1_	1600
	N_3_	2000
	N_5_	1230
	N_t_	2300
The velocity of sound (m/s)	V_d_	3300
	V_1_	3200
	V_3_	4000
	V_5_	2460
	V_t_	4600
Density (× 10^3^ kg/m^3^)	Ρ	7.5
Curie’s temperature (℃)	T_c_	300

The lower and upper discs are considered the input and the output ports respectively. In the experiments, two piezoelectric discs are placed concentrically and a weight is placed on them to provide the necessary force between them. It is expected that by stimulating the input piezoelectric disc in each “out-of-plane mode frequencies” of both piezoelectric discs, the output piezoelectric disc will vibrate in the radial mode. This phenomenon was observed and proven in experiments.

The input frequency is equal to the resonance frequency in the “out-of-plane mode” of a disc -each of the discs- and the output frequency is equal to the “radial mode” frequency of the outputting piezoelectric disc. The appearance of PFC is similar to a conventional transformer (PT), but it can increase the frequency. The piezoelectric discs dimension used in the simulation and experiment are shown in Table 2.

**Table 2:** Utilized piezoelectric elements dimensions.

Diameter (mm)	Thickness (mm)	D/T
20	2	10
28	2	14
50	2.9	17.2

## Simulation

The modal simulation of the piezoelectric disc was performed by COMSOL 6.0 to obtain the frequency of the out-of-plane modes of both elements and the radial mode of the output element. The simulation results for out-of-plane and radial modes are given in Tables 3 and 4. In Table 3, mode index refers to the number of diagonal nodes (n) and circular nodes (m). The mode index with n diagonal nodes and m circular nodes is displayed as (n, m).

**Table 3:** Mode shape, mode index, and out-of-plane resonance frequency (kHz).

	Index: (2,0)		Index: (0,1)
	f_20_ = 16.33		f_20_ = 32.47
	f_28_ = 8.50		f_28_ = 17.02
	f_50=_3.90		f_50_ = 7.82
	Index: (3,0)		Index: (4,0)
	f_20_ = 36.50		f_20_ = 61.14
	f_28_ = 19.42		f_28_ = 33.25
	f_50_ = 8.98		f_50_ = 15.53
	Index: (1,1)		Index: (5,0)
	f_20_ = 66.73		f_20_ = 89.09
	f_28_ = 36.03		f_28_ = 49.49
	f_50_ = 16.76		f_50_ = 23.34
	Index: (2,1)		Index: (0,2)
	f_20_ = 105.26		f_20_ = 115.51
	f_28_ = 58.39		f_28_ = 64.30
	f_50_ = 27.5		f_50_ = 30.34

The indies show the diameter of the piezoelectric disc.

**Table 4:** Mode shapes and radial resonance frequency (kHz), for piezoelectric discs of Table 2.

1st radial mode		2nd radial mode		3rd radial mode	
	${\mathrm{f}}_{\mathrm{r}20}$=114.23		${\mathrm{f}}_{\mathrm{r}20}$=292.69		${\mathrm{f}}_{\mathrm{r}20}$=447.39
	${\mathrm{f}}_{\mathrm{r}28}$=81.75		${\mathrm{f}}_{\mathrm{r}28}$=211.49		${\mathrm{f}}_{\mathrm{r}28}$=329.24
	${\mathrm{f}}_{\mathrm{r}50}$=45.81		${\mathrm{f}}_{\mathrm{r}50}$=118.82		${\mathrm{f}}_{\mathrm{r}50}$=186.53

In COMSOL, the mode partitioning factor translation along the z-axis was calculated for the out-of-plane modes of the three piezoelectric discs, and the absolute value of the results is given in Table 5.

**Table 5:** The amount of mode participation factor translation along the z-axis in out-of-plane frequencies (kHz).

D = 20 mm		D = 28 mm		D = 50 mm	
Frequency (kHz)	Participation factor	Frequency (kHz)	Participation factor	Frequency (kHz)	Participation factor
16.329	2.60E-13	8.5086	8.49E-12	3.9015	5.03E-11
32.469	1.68E-14	17.023	3.36E-12	7.8243	3.50E-12
36.501	7.39E-14	19.423	6.80E-13	8.9842	1.12E-11
61.145	2.59E-14	33.257	1.06E-12	15.532	3.34E-12
66.731	1.79E-15	36.034	8.22E-14	16.762	3.45E-13
89.09	2.09E-15	49.494	3.99E-13	23.348	9.94E-13
105.26	3.87E-15	58.398	3.58E-13	27.512	5.31E-14

## Experiments

**Figure 3:**
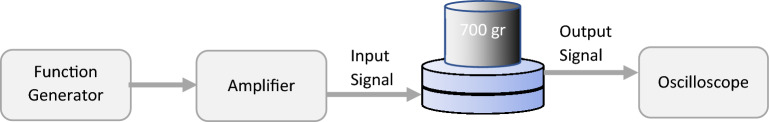
Test procedure.

Figure 3 shows the schematic of the components of a piezoelectric frequency converter system.

The piezoelectric discs are concentrically placed on each other and pressed together by a weight of 700 g. To grant the application of a uniform and perpendicular force to the nodes, a bullet is used. The force must be enough to ensure full contact and proper vibration transfer without noise. Also, the overweight reduces the vibration amplitude, and in practice, makes the device a single unit which is not proper.

**Figure 4:**
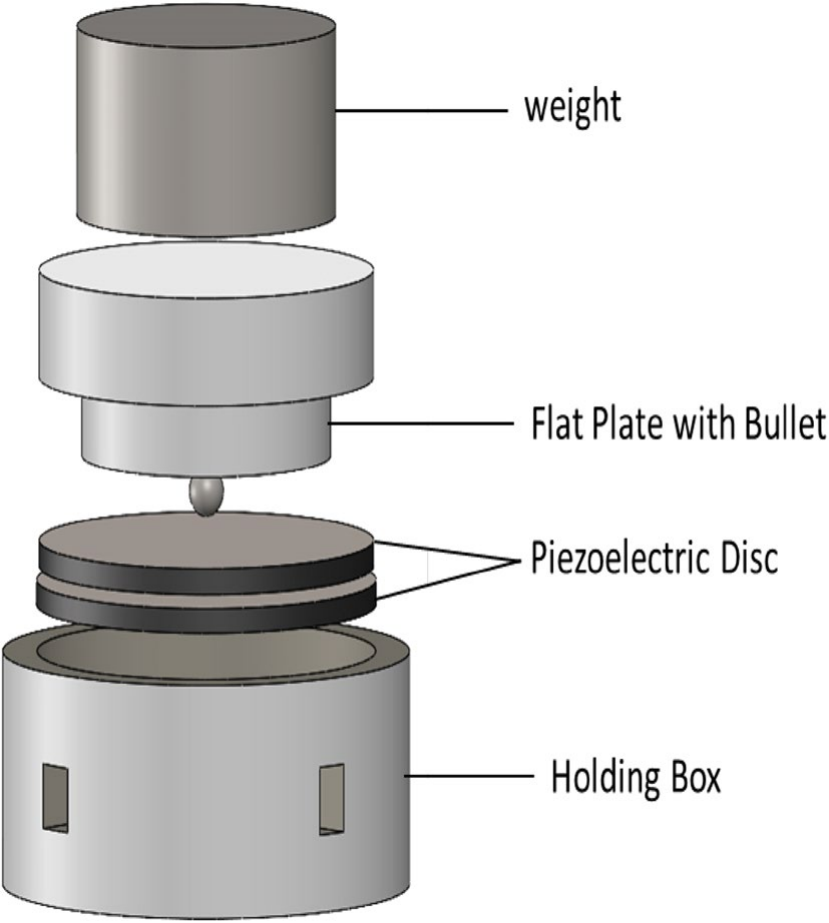
Exploded schematic of the experimental setup.

Figure 4 shows the exploded schematic of the test setup. The inner diameter of the chamber should be large enough to allow the piezoelectric discs to vibrate radially and at the same time keep them concentric. The difference between diameters is considered to be 0.25 mm. By placing a ring around smaller piezoelectric discs, they can be placed concentrically. Piezoelectric discs are connected in series. Electrodes made of thin copper foil were placed in the piezoelectric discs' lower, middle, and upper parts. The desired frequency is generated by a function generator, and after amplification is applied to the input port.

**Figure 5:**
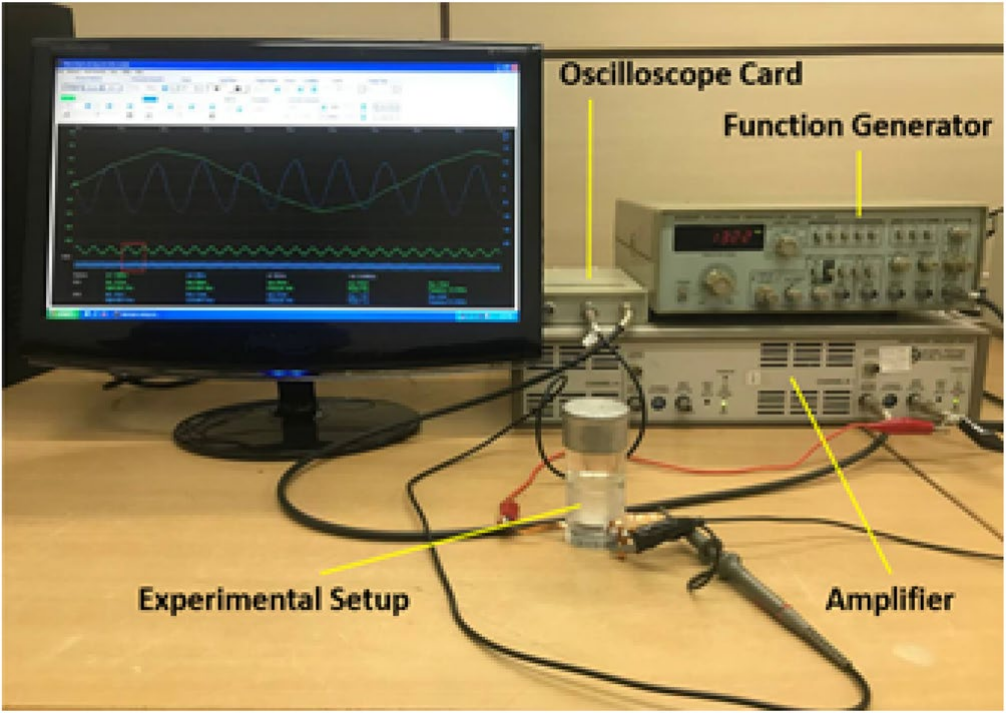
Experimental setup.

Figure 5 shows the experimental setup and related equipment.

**Figure 6:**
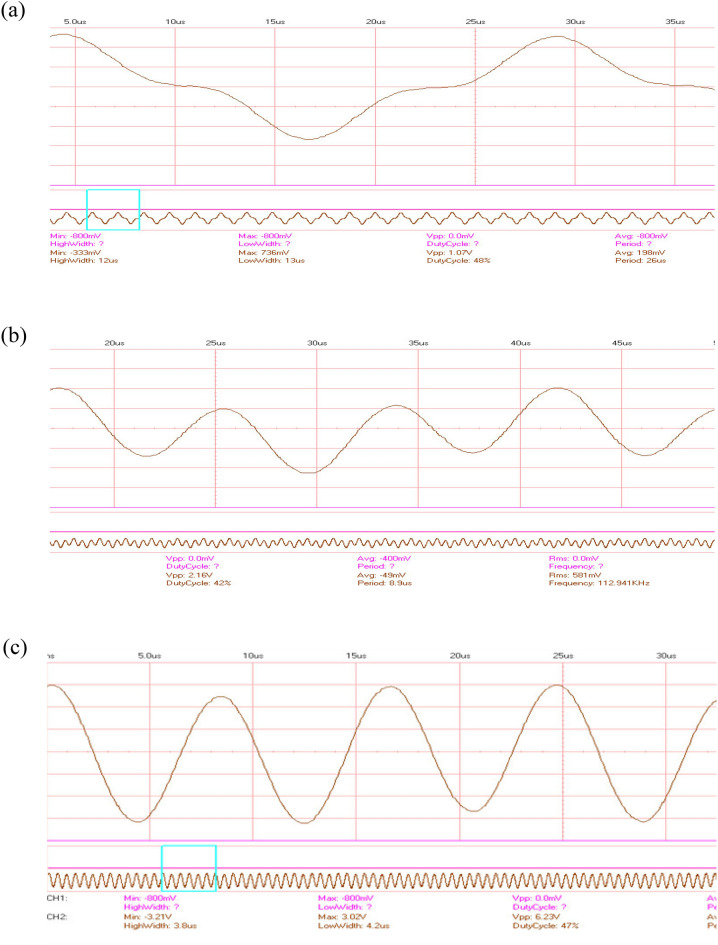
The output frequency for out-of-plane input frequency.

Figure 6a shows the stimulation of the input piezoelectric disc near each resonance frequency of the out-of-plane mode. The interference of the input and output waves first occurs, and as the frequency gets closer to the resonance frequency, the amplitude of the radial mode (output frequency) increases (Fig. 6b). Finally, only the radial mode frequency remains in the output (Fig. 6c).

**Figure 7:**
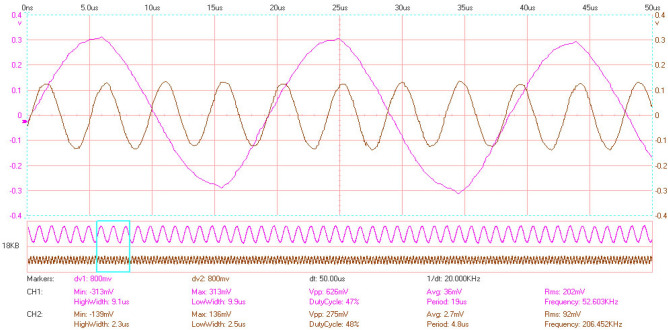
Output frequency for a pair of 28 mm discs and the input frequency of 52.6 kHz.

Table 6 shows the output frequencies related to out-of-plane input excitation frequencies. In the first three cases, similar discs are paired, and in the fourth case, discs with different diameters are paired. Figure 7 shows the test results for the input frequency of 52.6 kHz and the output frequency of 207 kHz.

**Table 6:** Test results for different pairs (D1 and D2 are input and output discs diameter).

D_1_ = 20 mm, D_2_ = 20 mm			D_1_ = 28 mm, D_2_ = 28 mm			D_1_ = 50 mm, D_2_ = 50 mm			D_1_ = 20 mm, D_2_ = 28 mm		
${\mathrm{f}}_{\mathrm{in}}$	${\mathrm{f}}_{\mathrm{out}}$	Gain	${\mathrm{f}}_{\mathrm{in}}$	${\mathrm{f}}_{\mathrm{out}}$	Gain	${\mathrm{f}}_{\mathrm{in}}$	${\mathrm{f}}_{\mathrm{out}}$	Gain	${\mathrm{f}}_{\mathrm{in}}$	${\mathrm{f}}_{\mathrm{out}}$	Gain
16	118	7.3	8.4	90	10.7	3.7	51	13.7	8.3	90	10.8
32.5	300	9.2	16.9	90	5.3	7.2	51	7.1	15.5	90	5.8
37	118	3.1	18.6	90	4.8	9.8	51	5.2	18.1	90	4.9
61.9	118	1.9	31.6	90	2.8	13.9	51	3.6	19.7	90	4.5
66.65	300	4.5	35.2	207	5.9	16.5	120	7.2	32.6	90	2.7
91.7	300	3.2	46.3	90	1.9	24.6	120	4.8	33.4	90	2.6
98	300	3.06	52.6	207	3.9	27.5	120	4.3	35.9	90	2.5

The radial mode frequencies of piezoelectric discs are measured with the impedance analyzer and the results are shown in Table 7.

**Table 7:** Radial mode frequencies (kHz).

Piezoelectric disc diameter	Mode	Impedance analyzer	Simulation	Experiment
20	1st	111.46	114.2	130
	2nd	288.54	292.08	300
	3rd	436.98	443.37	400
28	1st	80.21	81.74	90
	2nd	205.21	211.3	207
	3rd	322.4	329.24	313
50	1st	46.66	45.81	52
	2nd	121.33	118.82	120
	3rd	191.33	186.53	193

**Figure 8:**
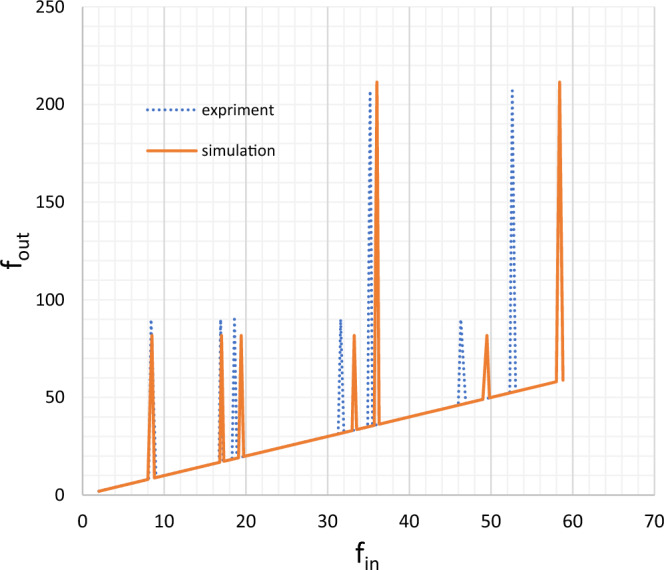
Input and output frequencies for the pair of 28 mm piezoelectric discs (kHz).

Figure 8 shows the simulation and test results for input and output frequency for a 28 mm piezoelectric discs pair. The bottom line shows the output and input frequencies are usually the same. For example, an input frequency of 40 kHz results in an output frequency of 40 kHz. But at the points where the peak is seen, the output frequency shows an increase. For example, an input frequency of 8.4 kHz leads to an output frequency of about 90 kHz. In this research, these peaks are used.

**Figure 9:**
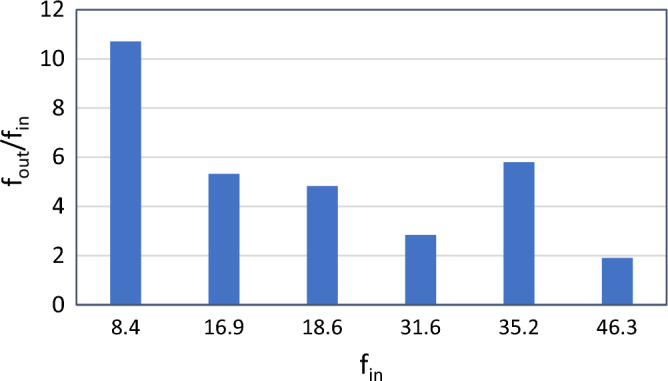
Gain diagram for a pair of 28 mm piezoelectric discs.

Figure 9 shows the graph of the ratio of output to input frequency in terms of input frequency for a 28 mm piezoelectric disc pair. In this figure, the first 4 input frequencies excite the first radial mode, so the frequency gain continues to decrease. But in the 5th mode, the frequency ratio increases, as the output excites the second radial mode.

**Figure 10:**
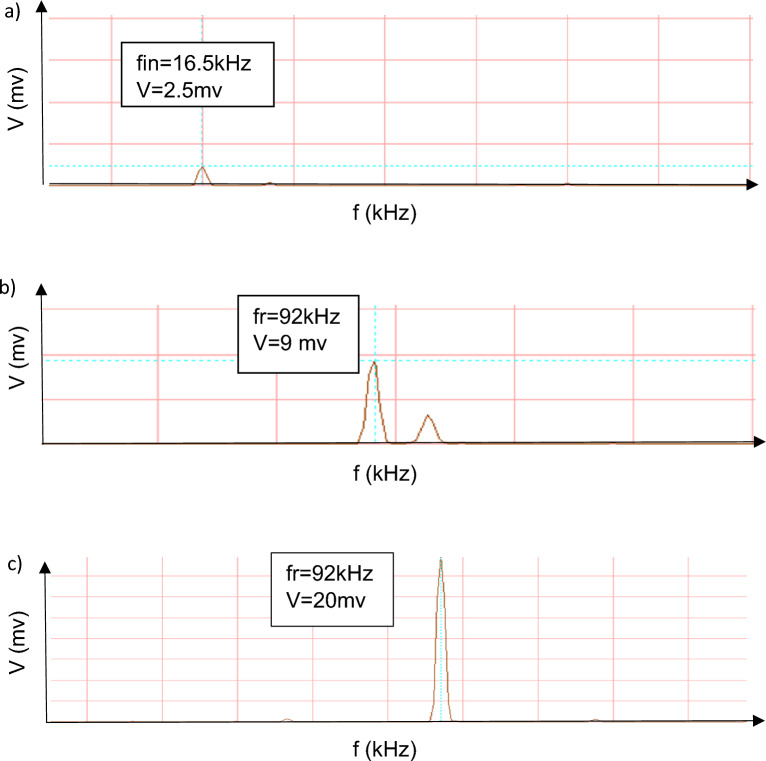
FFT results.

The mechanism of converting input frequency to output frequency can be considered as follows. The piezoelectric disc excitation in the out-of-plane mode due to the larger vibration amplitude in the z direction causes an impact to the output piezoelectric disc and excites all its natural modes. As a result, in addition to the forced vibration (input frequency), free vibration also occurs in the output piezoelectric disc and Radial natural modes, which have a larger amplitude than the forced vibration in the out-of-plane mode, are observed at the output, and an increase in frequency occurs in the out-of-plane modes. In this research, we used the radial mode with a larger amplitude as the output, while the next radial mode can be converted to the output with a filter and obtain a higher conversion factor. The FFT of the frequency increase phenomenon in the out-of-plane mode is shown in Fig. 10a. The input piezoelectric disc excitation near the out-of-plane mode excites all the natural modes, and the radial mode too, which the radial mode has a larger amplitude than other modes (Fig. 10b). By increasing the excitation frequency to the out-of-plane mode, the amplitude of the radial mode increases, and this mode is observed at the output frequency (Fig. 10c).

To compare the energy transferred in the z-direction, the data of Table 5 were rearranged in descending order of the mode participation z-translation factor for test and simulation (Table 8). Reducing the mode participation factors drives the output to a higher radial mode. In this research, the radial mode which has the larger amplitude is considered as the output.

**Table 8:** Mode participation factor reduction.

Out-of-plane frequency (kHz) simulation	Input frequency (kHz) experiment	Mode participation factor-z translation	Output radial mode no. experimental	Out-of-plane frequency simulation (kHz)	Input frequency (kHz) experiment	Mode participation factor-z translation	Output radial mode no. experimental
D_1_ = 20 mm, D_2_ = 20 mm				D_1_ = 28 mm, D_2_ = 28 mm			
16.329	16	2.60E-13	1	8.5086	8.4	8.49E-12	1
36.501	37	7.39E-14	1	17.023	16.9	3.36E-12	1
61.145	61.9	2.59E-14	1	33.257	31.6	1.06E-12	1
32.469	32.5	1.68E-14	2	19.423	18.6	6.80E-13	1
105.26	98	3.87E-15	2	49.494	46.3	3.99E-13	1
89.09	91.7	2.09E-15	2	58.398	52.6	3.58E-13	2
66.731	66.65	1.79E-15	2	36.034	35.2	8.22E-14	2
D_1_ = 50 mm, D_2_ = 50 mm				D_1_ = 28 mm, D_2_ = 20 mm			
3.9015	3.7	5.03E-11	1	8.5086	8.3	8.49E-12	1
8.9842	9.8	1.12E-11	1	17.023	18.1	3.36E-12	1
7.8243	7.2	3.50E-12	1	33.257	33.4	1.06E-12	1
15.532	13.9	3.34E-12	1	19.423	19.7	6.80E-13	1
23.348	24.6	9.94E-13	2	16.329	15.5	2.60E-13	1
16.762	16.5	3.45E-13	2	36.034	35.9	8.22E-14	1
27.512	27.5	5.31E-14	2	32.469	32.6	1.68E-14	1

## Comparing experiments and simulation

The maximum and minimum difference between the input frequency of the experiment and the simulation was calculated for a piezoelectric disc pair. The highest difference for three tests with a similar piezoelectric disc pair with diameters of 20, 28, and 50 mm was 10.5, 9.9, and 2.9 percent and the lowest difference was 0.04, 0.7, and 0.09 respectively. Piezoelectric disc pairs with different diameters of 20 and 28, had the highest difference of 6.3 and the lowest difference of 0.3 percent. Therefore, with the increase of the D/T ratio, the input frequency difference increases. The largest difference between the output frequency of the test and simulation, in the first radial mode, for similar piezoelectric disc pairs with diameters of 20, 28, and 50 mm is 3.3, 10, and 11 percent respectively and the lowest frequency difference is 2.4, 2 and 0.9, respectively. This difference cannot necessarily be considered an "error", because the characteristics between the piezoelectric disc in the test and the simulation may not be the same.

## Application

In this research, a frequency converter using two piezoelectric discs is presented, in which by choosing the appropriate piezoelectric discs, it is possible to make a frequency converter with the desired conversion ratio. In this research, investigation up to the second radial mode has been done, therefore the maximum accessible conversion ratio is 13.7. To increase this frequency ratio, the next radial mode can be considered as the output. A proper filter may be necessary to pick up the desirable frequency, among the other output frequencies. Also, the excitation voltage range was between 50 and 200 V, and the output voltage range was obtained between 0.4 and 4 V. Using an amplifier, the output voltage range can be increased to the desired level. In this converter, the excitation voltage does not affect the output frequency and the output frequency is only a function of the material, dimensions, and piezoelectric disc vibration mode.

In thin discs, the excitation frequency depends on the aspect ratio (D/T), and with the increase of this ratio, the number of out-of-plane modes increases. Therefore, by choosing the D/T ratio, the output frequency and desired excitation can be determined.

**Figure 11:**
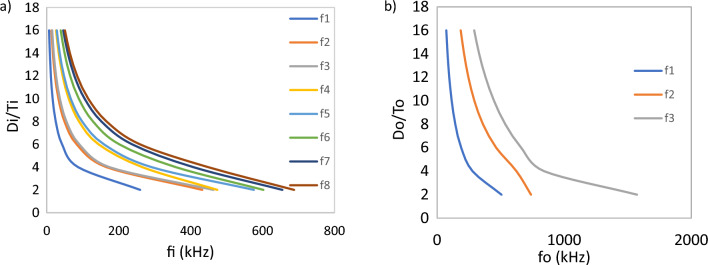
Effect aspect ratio (D/t) on out-of-plane (**a**) and radial (**b**) resonance frequency.

Figure 11a shows the simulation result of changes in out-of-plane frequencies relative to D/T and Fig. 11b shows the trend of radial frequencies of a piezoelectric disc relative to D/T at T = 2 using the finite element method.

To convert the f_1_ frequency to f_2_ frequency, we first obtain the (D/T)_1_ for the input piezoelectric disc from the diagram in Fig. 11a for desired f_1_. Then, from the diagram in Fig. 11b, we obtain the (D/T)_2_ ratio for the desired f_2_. There is more than one option for each ratio, and we can choose the most suitable among them. In the first case, we can assume the same thickness for both discs and obtain their diameters from the aspect ratio. In the second case, we can assume similar diameters and obtain the thicknesses of the discs.

## Conclusion

In this article, a new type of frequency converter called piezoelectric frequency converter (PFC) is introduced. The transducer consists of two piezoelectric discs, which are pressed together in a housing. Input stimulation in each of the out-of-plane modes of each piezoelectric disc causes the stimulation of the output piezoelectric disc radial mode. Modal analysis is done to confirm the resonance frequencies and mode shape. A few test samples were made and the performance of the piezoelectric frequency converter was tested. The experimental resonant frequencies are in good agreement with the simulated frequencies.

The conversion ratio depends on the out-of-plane modes of both the piezoelectric disc and radial modes of the output piezoelectric disc. These modes can be selected by choosing the diameter and thickness of piezoelectric discs, and the converter can be made with any desired conversion ratio. Therefore, by changing the D/T ratio, the input and output frequency can be specified.

Based on the obtained results, it can be concluded that PFC has a high potential to be used in applications that require an increase in frequency.

## Data Availability

All data generated or analyzed during this study are included in this published article.
